# Constitutive Nucleosome Depletion and Ordered Factor Assembly at the *GRP78* Promoter Revealed by Single Molecule Footprinting

**DOI:** 10.1371/journal.pgen.0020160

**Published:** 2006-09-22

**Authors:** Einav Nili Gal-Yam, Shinwu Jeong, Amos Tanay, Gerda Egger, Amy S Lee, Peter A Jones

**Affiliations:** 1 Department of Urology, USC/Norris Comprehensive Cancer Center, Keck School of Medicine, University of Southern California, Los Angeles, California, United States of America; 2 Department of Biochemistry and Molecular Biology, USC/Norris Comprehensive Cancer Center, Keck School of Medicine, University of Southern California, Los Angeles, California, United States of America; 3 Center for Studies in Physics and Biology, Rockefeller University, New York, New York, United States of America; Lawrence Livermore National Laboratory, United States of America

## Abstract

Chromatin organization and transcriptional regulation are interrelated processes. A shortcoming of current experimental approaches to these complex events is the lack of methods that can capture the activation process on single promoters. We have recently described a method that combines methyltransferase M.SssI treatment of intact nuclei and bisulfite sequencing allowing the representation of replicas of single promoters in terms of protected and unprotected footprint modules. Here we combine this method with computational analysis to study single molecule dynamics of transcriptional activation in the stress inducible *GRP78* promoter. We show that a 350–base pair region upstream of the transcription initiation site is constitutively depleted of nucleosomes, regardless of the induction state of the promoter, providing one of the first examples for such a promoter in mammals. The 350–base pair nucleosome-free region can be dissected into modules, identifying transcription factor binding sites and their combinatorial organization during endoplasmic reticulum stress. The interaction of the transcriptional machinery with the *GRP78* core promoter is highly organized, represented by six major combinatorial states. We show that the TATA box is frequently occupied in the noninduced state, that stress induction results in sequential loading of the endoplasmic reticulum stress response elements, and that a substantial portion of these elements is no longer occupied following recruitment of factors to the transcription initiation site. Studying the positioning of nucleosomes and transcription factors at the single promoter level provides a powerful tool to gain novel insights into the transcriptional process in eukaryotes.

## Synopsis

Control of gene expression and transcription are complex and well-coordinated processes. Most current experimental approaches to understanding the underlying mechanisms, which include binding of transcription factors to regulatory regions of genes, and changes in the structure and composition of chromatin, rely on studies of populations of cells and cannot capture the transcription activation process on single promoters. The authors describe the use of a footprinting method which enables analysis of chromatin structure and binding of factors on single DNA molecules. This is applied to study the activation process of GRP78, a protein which is important for the induction of a response to endoplasmic reticulum stress. By combining the footprinting method and computational analyses, the authors define functional modules on the GRP78 promoter and show that it exists in few major combinatorial states, reflecting its high level of organization. These results provide novel insights into the activation of GRP78 which could not be gleaned using conventional methods. They also demonstrate the use of the method as a unique and powerful tool to study the transcriptional process in eukaryotes, which remains a major source of interest and challenge for the scientific community.

## Introduction

The essential role of chromatin structure and organization in transcriptional regulation has been well established. This structure is mainly determined by the state of nucleosomes—the primary repeating units of chromatin. Recent experimental advances have provided a wealth of information contributing to the notion that nucleosomes are dynamic structures, able to change both their compositions and positions on DNA. Specifically, nucleosomes found at gene promoters are known to be remodeled by various complexes or disassembled, and the histones comprising them covalently modified, or replaced by variants in order to allow transcription to take place ([1], reviewed in [2]). An emerging concept arising from recent studies performed in yeast and flies is that nucleosome depletion at active promoters is a genome-wide phenomenon [3–6]. Specific examples in yeast include inducible genes such as *PHO* and heat shock proteins (HSPs) as well as constitutively expressed genes such as the housekeeping *GCY1* and *AKY2* genes [7–10]. In mammals, very few examples exist: reversible nucleosome depletion was demonstrated upon activation at the inducible *IL2* promoter, [9], while the enhancer of the *INF*-β gene was shown to be constitutively depleted of nucleosomes [11]. However, evidence on nucleosome depletion in constitutively expressed genes is lacking.

The causal and temporal relations between the processes of covalent histone modifications, nucleosome remodeling, and nucleosome depletion are only partially understood and different models exist for different genes. However, it is clear that these processes are interrelated with the dynamics of specific transcription factors and the general transcription machinery on the cognate promoters. A shortcoming of current experimental approaches to these complex processes is the lack of methods that can capture the activation process on single promoters. Current methods typically depict the dynamics of one relevant factor (e.g., nucleosome, transcriptional activator) for a population of cells in each experiment, making it difficult to understand the underlying process, which is orchestrated by a combination of several interrelated interactions.

We have recently described a footprinting method which allowed us to analyze chromatin structure on individual CpG-rich DNA molecules [12]. This was achieved by treating intact nuclei with the CpG-specific DNA methyltransferase M.SssI, which is able to methylate all CpG sites provided they are not protected by nucleosomes or by association with tight binding factors [12–14]. Following M.SssI treatment, DNA extraction, and genomic bisulfite sequencing of single clones, specific footprints can be revealed correlating with nucleosome positions and transcription factor binding on the studied promoters ([12], this study). An essentially similar method using methyltransferase HhaI in yeast has recently been described by others [15].

Here we extended the method (termed methylase-based single promoter analysis [M-SPA]) and combined it with computational analysis to study high-resolution patterns of DNA protection at promoters. The advantage of this approach over currently used footprinting methods lies in the combination of “positive marking” with single molecule resolution. Each sequence provides footprint information for one promoter molecule—a single functional unit. Studying a pool of such units enables one to analyze and detect linkage among the footprints generated and infer the dynamics of the studied region.

The unfolded protein response is conserved from yeast to human, triggering multiple pathways to allow cells to adapt to stress targeting the endoplasmic reticulum (ER) [16]. The ER is an essential organelle for the synthesis and folding of secretory and membrane proteins and is also a major intracellular calcium store. The glucose-regulated protein GRP78 (also referred to as BiP) is an evolutionary conserved molecular ER chaperone which plays a critical role in the maintenance of ER homeostasis [17]. While being ubiquitously expressed at a basal level in various cells, it can be highly induced (as much as 20-fold) as a consequence of a variety of stress conditions that perturb ER function and homeostasis [16]. This induction, widely used as an indicator of ER stress [18], was shown to be primarily mediated by three copies of the ER stress response elements (ERSEs), located upstream of a TATA element on the promoter [19,20]. ERSE binding factors include NF-Y, YY1, TFII-1, Sp transcription factors, and the nuclear form of ATF6 [19,21–25]. NF-Y binds constitutively to the promoter, while ATF6 is activated by ER stress and, acting in concert with YY1, is important in its induction upon stress [23,26].

The promoter of the *GRP78* gene is embedded in a CpG island, providing a dense CpG grid that enabled us to apply M-SPA in order to study the dynamics of its chromatin structure and functional organization during stress induction. Using this method to examine 294 promoter replicas, we find that a minimal region of approximately 350 base pairs (bp) of this promoter, encompassing the TATA box and transcription initiation site (TIS), is constitutively devoid of nucleosomes regardless of its induction state. Furthermore, we were able to dissect the *GRP78* promoter into functional modules correlating to the TATA box, TIS, and ERSEs and to study their combinatorial organization during stress induction. This showed that these modules are linked in a highly controlled fashion, revealing six major promoter states reflecting transcription factor loading and recruitment of the RNA polymerase machinery. Our results provide one of the first examples of constitutive nucleosome depletion from a mammalian inducible promoter and demonstrate the use of M-SPA as a powerful tool to study combinatorial transcription dynamics using single promoter entities.

## Results

### The *GRP78* Core Promoter Is Devoid of Nucleosomes

**Figure 1:**
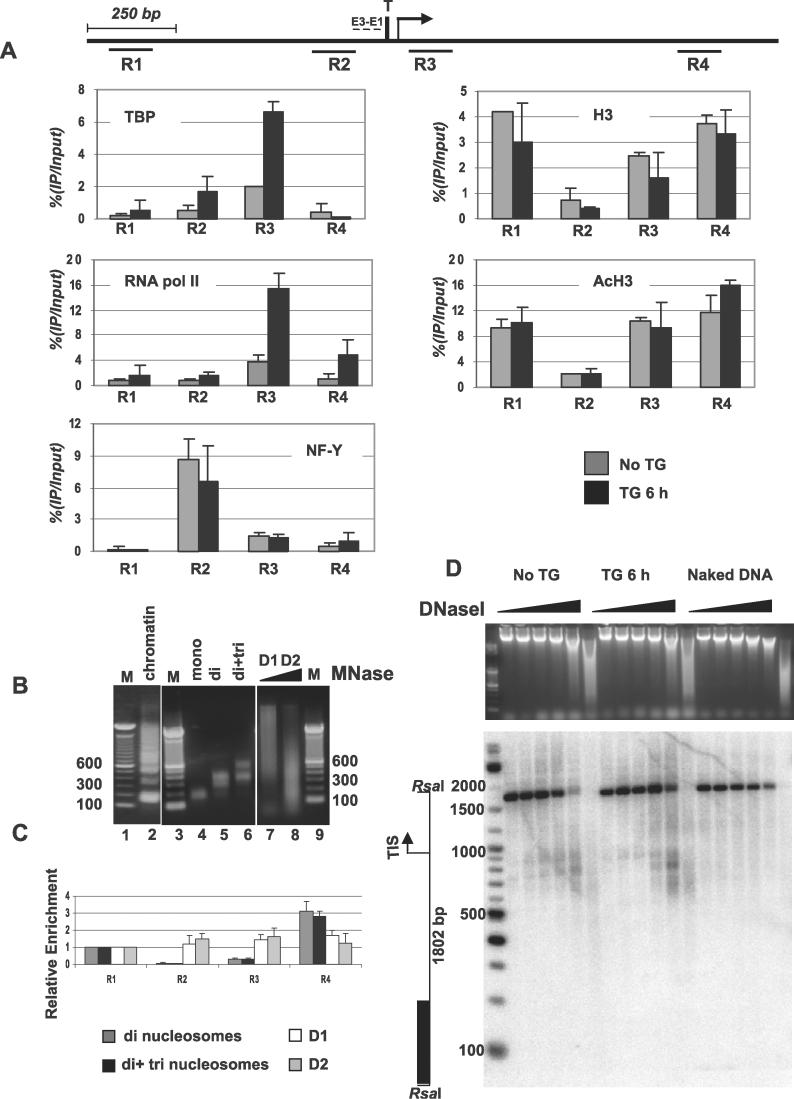
Transcription Factor Binding and Nucleosome Depletion at the Human *GRP78* Promoter (A) ChIP analysis of the *GRP78* promoter was performed on noninduced or TG-induced LD419 cells using antibodies against TBP, RNA polII, NF-Y, total H3, and acetylated H3-K9/14. Precipitated DNA was quantified by real-time PCR using primers specific for the indicated four regions of the promoter. The enrichment at each region is plotted as percentage of input. The data are representatives of experiments performed from two or three independent chromatin preparations. (B, C) MNase assay. (B) A mixture of oligonucleosomes (lane 2) from LD419 nuclei digested with MNase was fractionated through a sucrose gradient to obtain mononucleosomal, dinucleosomal, and trinucleosomal DNA (lanes 4, 5, and 6). Purified genomic DNA partially digested with MNase was used as naked DNA control (D1 and D2, lanes 7 and 8; M in lanes 1 and 9 indicates size marker). (C) Relative enrichment of nucleosomal and naked DNA at four regions of the *GRP78* promoter. The values plotted are normalized with respect to the value at the R1 region, arbitrarily defined as 1. The four regions are depicted on the promoter diagram at the top of the figure. The TATA box (T), TIS (bent arrow), and ERSEs (E1–E3) are marked. (D) DNase I hypersensitivity assay. Top: EtBr staining. Bottom: Southern blot. The assay was performed on nuclei extracted from noninduced or TG-induced cells. Genomic DNA was included as a control to show a lack of sequence specificity of the enzyme. DNase I–digested samples were resolved by gel electrophoresis, showing varying extents of digestion (EtBr staining). Southern blot performed after RsaI digestion revealed a hypersensitive region in the *GRP78* promoter similar in size in both noninduced and induced samples. On the left, a map of the GRP78 promoter region is shown indicating the 1,802-bp DNA fragment generated by RsaI digestion, transcription start site (bent arrow), and probe fragment (black box). Numbers next to the marker indicate size in bp.

The evolutionary conserved *GRP78* gene is constitutively expressed at a basal level in most cells and can be highly induced upon various stress signals. The most commonly used is treatment with thapsigargin (TG), which inhibits the ER calcium ATPase pump and depletes ER calcium stores [27]. The promoter of the gene is embedded in a CpG island and contains a TATA box (Figure 1), an unusual feature for CpG island promoters. This organization proved ideal for our studies in that it provided both a dense CpG grid and one defined transcription initiation site. First, we performed several population-based studies on either basally expressing cells or cells that were stress induced by TG treatment for 6 h, resulting in an approximately 10-fold increase in *GRP78* mRNA levels (data not shown). Chromatin immunoprecipitation (ChIP) assays using antibodies against TATA-binding protein (TBP) and RNA pol II revealed enrichment of these factors on the promoter at the region (R3) near the TIS (Figure 1A). This enrichment was already apparent at the zero time point, indicating the association of these factors before induction (compare enrichment of both factors at R3 to enrichment at R1, 750 bp upstream of the TIS). After stress induction, both factors were further recruited to the promoter as was also recently shown by others [28]. ChIP assays using antibodies against the transcription factor NF-Y, which constitutively resides on the promoter [21,28], revealed enrichment at the ERSE region which did not significantly change after stress induction (R2, Figure 1A). Analysis of the chromatin structure of the promoter region using antibodies that recognize total histone H3 (H3) and acetylated histone H3 (AcH3) revealed that H3 and its modified form are clearly depleted in the region upstream of the TATA and TIS regions (R2) compared with a higher enrichment (approximately 3.5-fold) in the region just downstream of the TIS (R3) and to a yet higher fold (approximately 6-fold) at regions 1 and 4 which are found 750 bp upstream and 830 bp downstream of the TIS, respectively (Figure 1A, right). No significant changes in either H3 occupancy or H3 acetylation were seen after stress induction. Similar depletion at R2 was observed for the H3K4-trimethyl mark (data not shown). The pattern of H3 depletion at R2 resembles the “5′ DIP” pattern shown by others at active yeast and *Drosophila* promoters [6,29].

To validate that these results reflected nucleosome depletion, we examined the sensitivity of the various regions of the promoter to MNase. Basally expressing LD419 nuclei were treated with MNase at a concentration that yielded a mixture of molecules containing nucleosomal repeats of various lengths (Figure 1B, lane 2). This mixture was run on a sucrose gradient to separate fractions enriched in oligonucleosomes (Figure 1B, lanes 4–6). The DNA from the fractions enriched in dinucleosomes and trinucleosomes was then extracted and subjected to quantitative PCR analysis using the same primer sets used for the ChIP assay (Figure 1C). We reasoned that the area depleted from nucleosomes according to the ChIP analysis should also be hypersensitive to MNase and thus yield a significantly lower PCR product when oligonucleosomal DNA was used as template. Indeed, both fractions amplified were depleted to a high extent in R2 compared to R1 (approximately 12-fold) and R3 (approximately 5-fold), similar to the trend in the ChIP experiment (Figure 1C). The higher enrichment at R4 may reflect a well-positioned nucleosome at this region. These results do not reflect inherent hypersensitivity of the R2 region to MNase as similar real-time PCR analysis performed on naked DNA partially digested with MNase (Figure 1B, lanes 7 and 8) did not show a significant difference in enrichment between the four regions (Figure 1C). Finally, we performed a DNase I hypersensitivity assay on nuclei obtained from untreated and TG-treated cells. These were incubated with increasing amounts of DNase I to obtain suitable levels of digestion (Figure 1D, top). Southern blotting of the DNase I–treated samples revealed a region of hypersensitivity which was similar in size in both noninduced and induced cells. This region spanned approximately 400 bp and included the TIS, indicative of the absence of nucleosomes in this region (Figure 1D, bottom). The heterogeneity of the smear in the hypersensitive region probably reflects some protection to DNase I digestion by transcription factors bound on the promoter, which is consistent with our ChIP (Figure 1A) and M.SssI analyses (see below). Taken together, these results indicate that the *GRP78* core promoter is associated with both specific (NF-Y) and general (RNA pol II and TBP) transcription factors in both its basally expressing and induced states, and this is concomitant with nucleosome depletion at this region. However, these classic techniques cannot define the percentage of promoter molecules bound by transcription factors and those that are nucleosome depleted. They also do not show whether there is a linkage between transcription factor binding and nucleosome depletion.

### M-SPA Reveals a Constitutive 350-bp Nucleosome-Free Region on the *GRP78* Promoter

**Figure 2:**
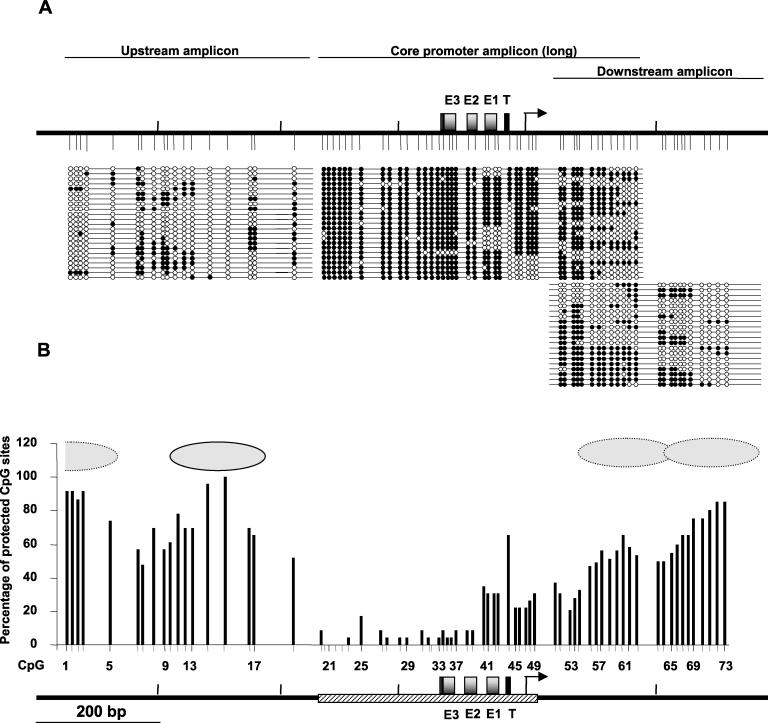
The Nucleosome-Free Region on the *GRP78* Core Promoter Is Minimally 350 bp Long (A) The diagram on top, drawn to scale, represents the region analyzed and indicates the distribution density of the 73 CpG sites (tick marks) included in this region. The position of the ERSEs (E1–E3), TATA box (T), and transcription initiation site (bent arrow) are indicated. Nuclei were extracted from actively growing LD419 cells, followed by 15 min M.SssI treatment, bisulfite conversion of extracted DNA, PCR amplification of three regions of the promoter covering 1100 bp, and cloning and sequencing of individual clones. Each horizontal line with a string of circles represents the methylation profile for one DNA molecule. White circles indicate unmethylated, and black circles, methylated CpG sites. (B) Summed protection levels at individual CpG sites. The striped bar represents the inferred nucleosome-free region. Note that the relatively high protection levels at CpGs 40 to 50 are contributed by distinct footprints which are too small to reflect nucleosomes. The ovals represent the predicted nucleosome positions (solid line, well positioned nucleosome; dotted line, less well positioned nucleosomes or no nucleosome boundary).

We exploited the fact that the *GRP78* promoter is embedded in a CpG island to study its chromatin structure and functional organization using M-SPA. In this method, intact nuclei are treated with M.SssI followed by DNA extraction, bisulfite conversion of the DNA, and PCR amplification of the studied region. The PCR products are cloned and single clones are sequenced, providing protection patterns for single promoter molecules [12]. Three amplicons covering an approximately 1,100-bp region of the *GRP78* promoter including a total of 73 CpG sites were initially analyzed. Bisulfite sequencing of naked DNA revealed that the *GRP78* promoter is totally unmethylated, as expected, which is a prerequisite for the use of this method (Figure S1A). Treatment of naked DNA with M.SssI for a 15-min period resulted in virtually 100% methylation, demonstrating the efficiency of M.SssI methylation at this region (Figure S1B). Analysis of sequences derived from nuclei extracted from noninduced cells treated with M.SssI revealed long patches of protected CpGs, spanning roughly 150 bp or longer, that are diagnostic for the presence of nucleosomes [12] (Figure 2A). In all sequenced molecules, we detected such nucleosome footprints upstream of the core promoter (Figure 2A, upstream amplicon), possibly reflecting a positioned nucleosome in this region. Downstream of the TIS, such long protected patches were present in many of the core promoter amplicon molecules and on all downstream region molecules, suggesting the existence of nucleosomes in these regions. However, these seemed to be less positioned. Remarkably, while small footprints were apparent, no 150-bp nucleosome protection patterns were found on an approximately 350-bp region upstream of and encompassing the TIS. These findings are in agreement with the ChIP, MNase, and DNase I data, and enabled us to delineate a minimal region of approximately 350 bp of the *GRP78* promoter which is devoid of nucleosomes (Figure 2B). This phenomenon was not unique to LD419 cells as nucleosome depletion from this promoter was also present in four other cell lines studied (RKO, HCT116, SW13, and A-427, data not shown).

### Dissection of the *GRP78* Promoter Defines High-Resolution Footprint Modules

**Figure 3:**
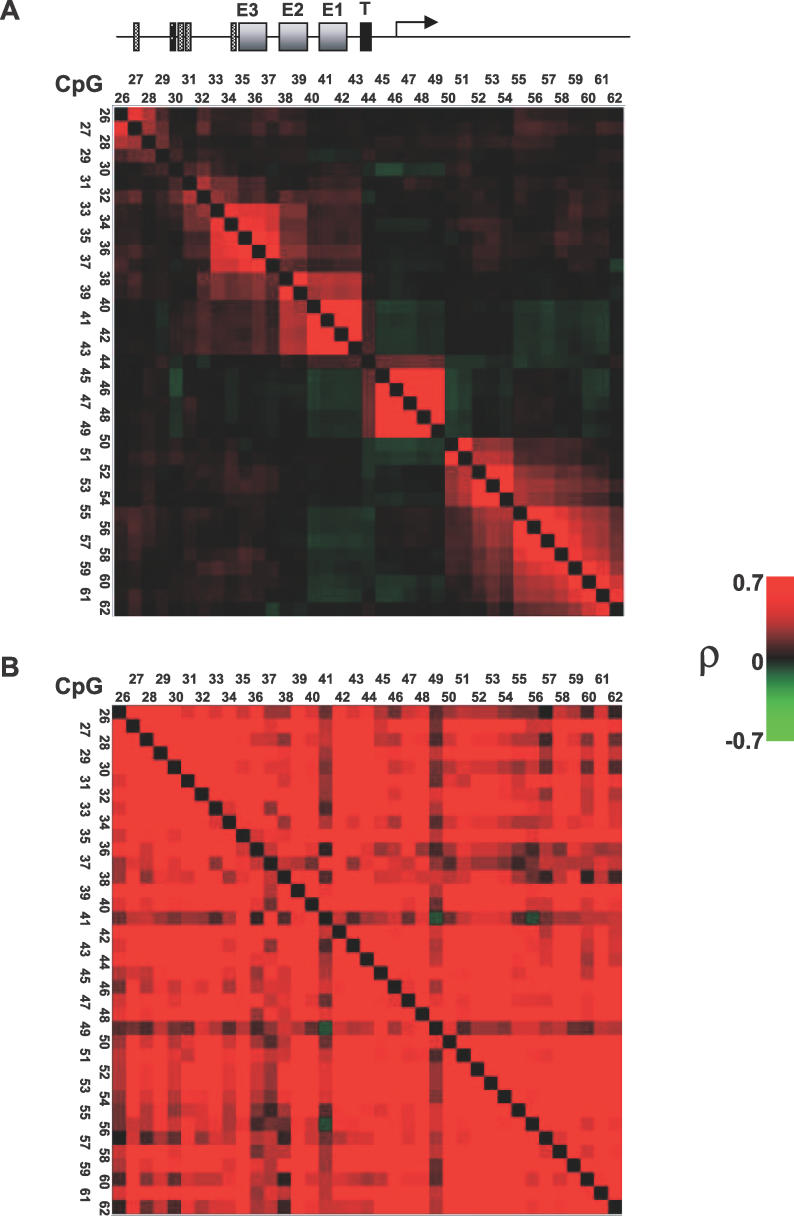
Definition of Footprint Modules in the Core Promoter (A) Shown is a color-coded representation of the correlations among protection patterns of 37 CpGs in the *GRP78* core promoter region (CpGs 26–62). Red indicates positive correlation, and green indicates negative correlation. The evident red blocks on the diagonal correspond to contiguous sets of CpGs that are highly co-protected in the pool of 294 promoter sequences. Although discovered in a completely unsupervised fashion, these blocks correspond to distinct and known functional promoter elements. (B) A similar correlation analysis performed as a negative control on 40 sequences derived from naked DNA which was methylated to achieve 60% average methylation. No defined modules similar to those as in (A) are found. For both (A) and (B), note that the matrix is symmetric by definition; both upper and lower triangular parts are drawn for visualization purpose only.

**Figure 4:**
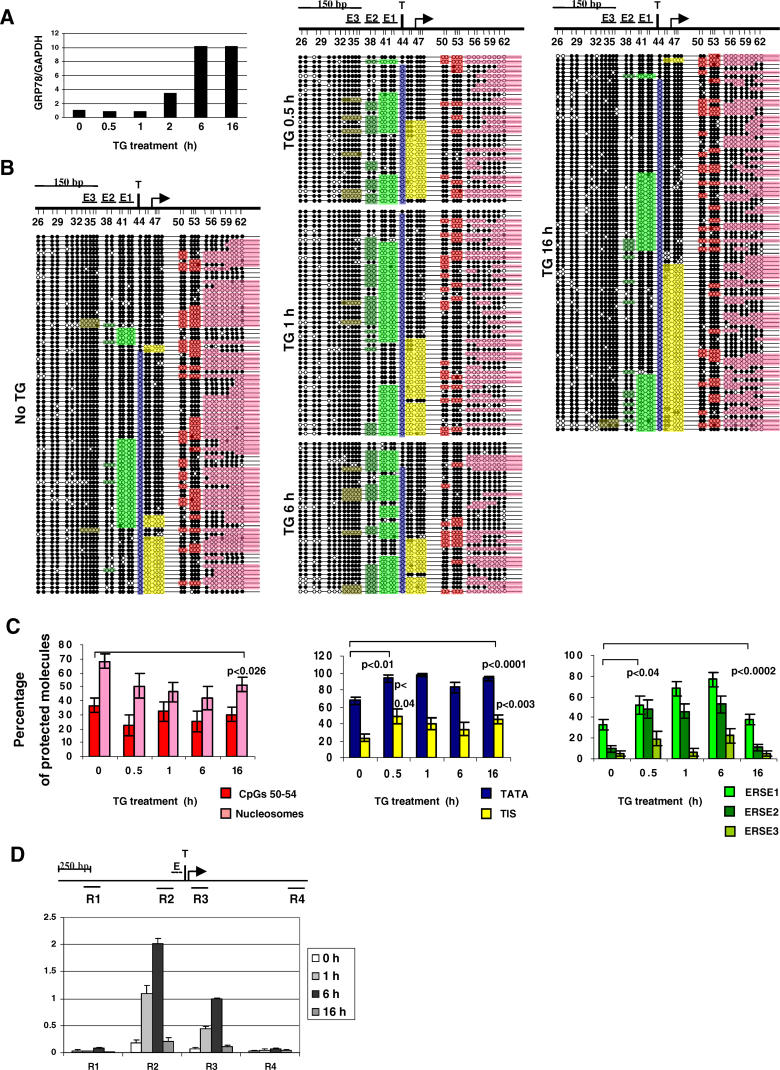
*GRP78* Activation during ER Stress LD419 cells were harvested at 0, 0.5, 1, 2, 6, and 16 h after induction with 300 nM TG. The nuclei were purified and treated with M.SssI followed by bisulfite genomic sequencing. (A) Relative *GRP78* mRNA levels at the different points after TG induction determined by quantitative RT-PCR (normalized to GAPDH). (B) Sequencing data for the various time points. The diagrams on top, drawn to scale, represent the region analyzed (core promoter amplicon, short) and indicate the distribution density of the 37 CpG sites included in this region. The TIS (bent arrow), TATA box (T), and ERSE elements (E1–E3) are marked. Each horizontal line with a string of circles represents the methylation profile for one DNA molecule. White circles indicate unmethylated, and black circles, methylated CpG sites. The modules, as defined by the correlation analysis in Figure 3, are color coded: blue, TATA box module; yellow, TIS module; bright green, ERSE1 module; dark green, ERSE2 module; olive, ERSE3 module; dark red, CpGs 49–50 and 51–53 modules; pink, nucleosomal module. The modules were colored if the majority of CpGs comprising the module were protected. The nucleosomal module was colored according to the previous nucleosomal patch definition [12] where more than two consecutive protected CpGs are considered a patch and the occurrence of one unmethylated CpG site does not break the contiguity of the patch. (C) Protection levels of nucleosomal modules (left), TATA and TIS (middle), and ERSEs (right) were calculated (see Materials and Methods) and are shown at the different time points. (D) ATF6 enrichment at the ERSE region shown by ChIP analyses on LD419 cells harvested at 0, 1, 4, and 16 h after TG stress induction. DNA was quantified by real-time PCR using primers specific for the indicated four regions of the promoter (as in Figure 1A).

While no nucleosomes were detected on the core promoter, several smaller protected regions, or footprints, were apparent in this region (Figure 2, core promoter amplicon), the most striking of which was a CpG site found just 2 bp downstream to the TATA box and protected in 16 of 23 molecules (69%). We therefore concentrated on a region which spans 495 bp around the TIS, including 37 CpG sites (CpGs 26–62, Figure 2), to study the dynamics of protection patterns at the *GRP78* promoter during ER stress induction. We used M-SPA to analyze 294 promoter sequences, derived from both basally expressing cells and cells harvested at various time points after TG stress induction (Figures 3 and 4).

First, we performed clustering analysis of CpG protection patterns on the pool of 294 sequenced molecules to reveal contiguous groups of highly co-protected CpGs which we define as “footprint modules” (Figure 3A). Modules represent CpGs that are protected in a similar fashion (either protected together or are unprotected together), suggesting that a common factor is bound to them in most cases. The modules that were defined by this analysis were surprisingly well correlated with known domains of the *GRP78* promoter (Figure 3A): The most upstream module (CpGs 26 and 27) correlates with a predicted Sp1 binding site [21]. Further downstream, three distinct footprint modules were defined (CpGs 33–37, CpGs 38–39, and CpGs 40–43) corresponding to the three conserved endoplasmic reticulum stress response elements (ESREs) [19,20]. The defined TIS [30], flanked by CpGs 45 to 49, was also strongly identified as a module. Downstream of the TIS, corresponding to the region where nucleosome patches were detected in many of the sequenced molecules (Figures 2A and 4B), a pronounced larger and “fuzzier” protection pattern was observed (CpGs 50–62). This module was further subdivided into two or three modules, possibly indicating additional binding of factors other than nucleosomes at the upstream submodules (CpGs 50–51 and 52–54). Therefore, in our further analyses we regarded the downstream submodule (CpGs 55–62) as reflective of nucleosomal protection (see Figure 4). To ensure that the defined modules were not generated by intrinsic properties of M.SssI, we performed the same clustering analysis on 40 sequences derived from naked DNA treated with M.SssI at a concentration that yielded a similar overall methylation level to the 294 pooled sequences (approximately 60%, see Figure S2 for the single sequence traces). While methylation was not completely random, no similar modules were defined on the naked DNA by this analysis (Figure 3B). Thus, using M-SPA we could systematically dissect the *GRP78* promoter into footprint modules, well supported to be functional by the known organization of the *GRP78* promoter, and our ChIP data (Figure 1A).

### Kinetics of Sequence Modules during GRP78 Stress Induction

We next analyzed the protection dynamics of the defined footprint modules during the time course of stress induction (Figure 4). An approximately 3.5-fold increase in *GRP78* mRNA levels was detected at 2 h after TG induction, reaching a maximum of 10-fold increase at 6 h which was maintained up to 16 h (Figure 4A). Analysis of the 294 sequences (Figure 4B) revealed that regardless of induction state, no nucleosomal patches were found upstream of the TIS, indicating that the 350-bp region defined in Figure 2 is constitutively nucleosome free. Downstream of the TIS, however, at the previously defined nucleosomal region (CpGs 55–62, see Figure 3), a change in protection pattern was apparent immediately after TG treatment (t = 0.5 h), where a decrease in nucleosomal patches was found (Figure 4B, pink patches, and 4C, left graph). This decrease, possibly reflecting sliding or remodeling of the nucleosomes driven by stress induction and ongoing transcription, was maintained up to the 16-h time point (Figure 4C, left graph, *p* < 0.026). Interestingly, no significant changes in protection levels were apparent for the upper submodules (CpGs 49–53) along the time course, again suggesting that these are bound by different factors other than those occupying the CpGs 55-to-62 submodule.

At the core promoter, CpG 44, near the TATA box, was already protected to a substantial level (67%) at the basal state—before stress induction. This protection significantly increased immediately after TG treatment (94% protection at t = 0.5 h, *p* < 0.01 (χ^2^ test)) and was retained at high levels throughout induction (t = 16 h, 93% protection, *p* < 0.0001, Figure 4B, blue bars, and 4C, middle graph). Protection of the TIS module followed a similar trend (increased protection at t = 0.5 h, *p* < 0.03, at t = 16 h, *p* < 0.003), however, with lower levels compared to TATA box protection (Figure 4B, yellow bars, and 4C, middle graph).

Interestingly, the dynamics of protection of the ERSE modules followed a different trend: All three ERSE footprints were induced at 30 min after treatment, albeit at different levels (Figure 4B bright green, dark green, and olive bars, and 4C, right graph). This protection increased along the time course (*p* < 0.0002 for t = 6 h) but decreased to nearly basal levels at 16 h after induction (*p* < 0.5). The pattern of protection at the ERSE region was recapitulated in a ChIP experiment using antibodies against the ERSE binding factor, ATF6, which is activated by ER stress and is important in the induction of GRP78 [26]. As shown in Figure 4D, ATF6 was enriched at the ERSE region at 1 h after induction, peaking at 6 h and decreasing to basal levels at 16 h after induction. However, the lower resolution of the ChIP compared to the M-SPA method could not discern between the multiple ERSE modules and their differential protection.

Taken together, these results reveal that factor loading and possibly chromatin remodeling take place shortly after induction, well before an elevation in mRNA levels is detected. They also reflect different binding kinetics for the general transcription machinery compared to specific transcription factors binding at the ERSEs.

### Few Discrete Promoter States Facilitate GRP78 Induction

**Figure 5:**
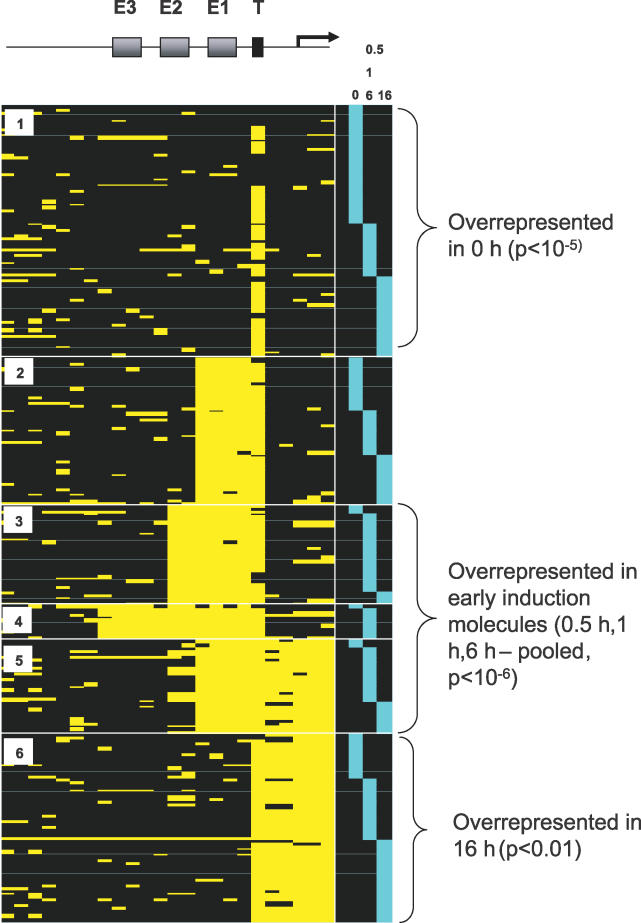
Few Combinatorial Modes of *GRP78* Promoter Organization Shown are clustered protection patterns for the 294 sampled promoters (rows, see Materials and Methods). Only few modes of promoter organization are observed, including clusters representing high levels of TATA binding (cluster 1), cassette like loading of the ESREs (clusters 2–4), recruitment of factors to the TIS (cluster 5), and release of the ESRE modules (cluster 6). Statistical enrichment analysis (Materials and Methods) confirms that specific modes of activity (clusters) are overrepresented in specific phases of the ER-stress activation process, enabling us to arrange the clusters in a chronological order. The designation of each row (= protection pattern of one promoter molecule) to the time point from which it originated is marked by the blue boxes on the right. The early induction time points (1, 0.5, and 6 h) are pooled.

The single molecule resolution afforded by M-SPA allowed us to examine possible linkage between the various footprint modules on the promoter. Looking at the correlation between nucleosomal region protection and transcription factors binding revealed that while at the population level stress induction resulted in an overall decrease of nucleosomal patches downstream of the TIS, at the single molecule level, no specific correlation existed between nucleosome positions and transcription factor binding (Figure 4B). In order to study the nucleosome-free region itself, we pooled data from all time points and performed an unsupervised clustering analysis of the protection patterns at CpGs 26 to 49, covering the TIS and the region upstream of it (Figure 5). Interestingly, all sequences were grouped into six defined clusters, suggesting that the *GRP78* core promoter exists in six major modes and that its organization is controlled by strong structural or mechanistic constraints. Cluster 1 represents promoters of which most, but not all, are TATA box protected, probably by TBP and other factors. Clusters 2, 3, and 4 represent cassette-like loading of transcription factors on the ESREs. As there is almost complete lack of protection of ERSE2 without 1 or protection of ERSE3 without 1 and 2, this may reflect sequential loading of these sites (from ERSE1 to ERSE3) during induction. Cluster 5 represents recruitment of the transcriptional machinery to the TIS, and in cluster 6, the TIS is protected while ERSEs are not.

**Figure 6:**
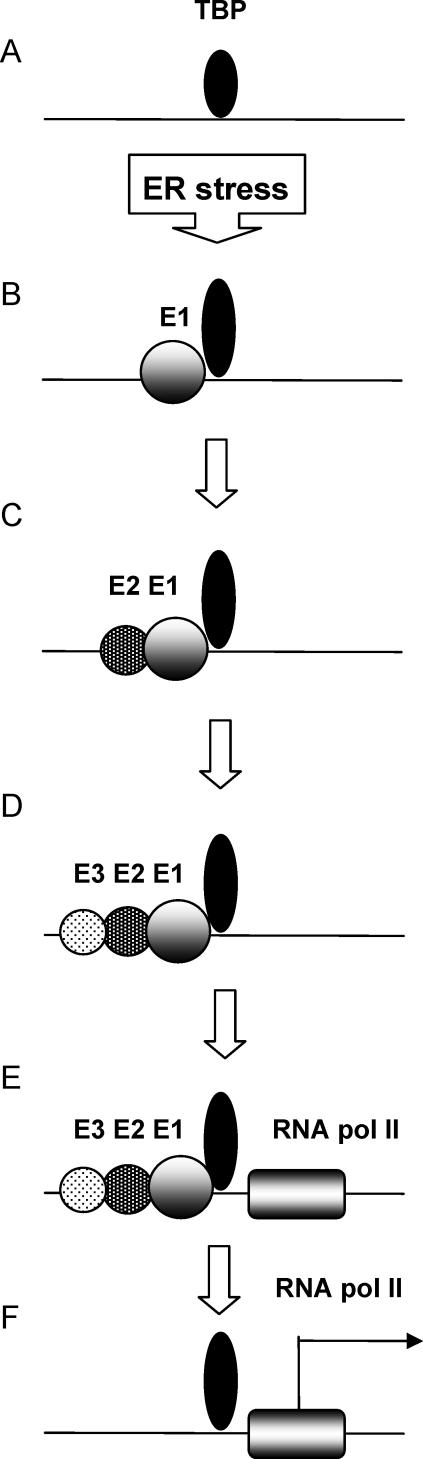
A Model Proposed for the Activation Process at the *GRP78* Promoter (A) The TATA box is occupied to a high extent even before induction, probably by TBP but also other factors. (B-D) After stress induction the ERSEs are sequentially loaded, from E1 to E3 while each ERSE is occupied at a different level (indicated by the size of the circles). (E, F) Recruitment of factors to the TIS is followed by release of the transcription factors from the ERSEs.

To test how these proposed states correlate with the time-course data, we analyzed the enrichment/deprivation of the various clusters in the preinduction (t = 0 h), early induction (t = 0.5 h, 1 h, 6 h), and late induction (t = 16 h) time points. This analysis revealed an overrepresentation of cluster 1 in the 0-h time point (*p* < 10^−5^, hypergeometric test), of clusters 3 to 5 in the pooled early induction time points (*p* < 10^−6^), and of cluster 6 in the 16-h time point (*p*<0.01). We also detected a matching underrepresentation of clusters 3 and 5 in the 0-h time point, of cluster 1 in the early induction time points, and of cluster 3 in the 16-h time point. The combination of the clustering data and the temporal enrichment analysis suggests that the *GRP78* core promoter switches between a small repertoire of states and enables us to determine the chronological order of these states, proposing a model for its transcriptional activation process (Figure 6). The possible mechanisms governing the transition between such states are discussed below.

## Discussion

Depletion of nucleosomes at promoters of active genes is considered a genome wide phenomenon in yeast and flies [3,5]; however, this has not been systematically studied in mammals and only sporadic cases have been published. For example, the human *IL*2 promoter was recently shown to be depleted of nucleosomes upon induction and these were then repositioned after removal of the inducing signal [9]. At the *INF*-β promoter, the enhancer was shown to be constitutively nucleosome depleted while a downstream nucleosome was remodeled to enable binding at the TATA box and TIS after induction [11]. Despite wide implications of ER stress and the unfolded protein response in development, health, and disease [17,31], the in vivo mechanism of ER stress activation of gene expression in the context of transcription factor binding and chromatin remodeling is just emerging [23,28]. Specifically, the kinetics of nucleosome binding and positioning on ER stress-regulated genes such as *GRP78* were not studied until now. Using several different methods, we show that the *GRP78* core promoter is constitutively depleted of nucleosomes at a region spanning 350 bp upstream and inclusive of the TIS, which could theoretically accommodate two nucleosomes. This is unambiguously exemplified by our sequencing data in which all 294 individual promoter molecules were virtually devoid of nucleosomal patches at this region regardless of the induction state of the promoter. This is different from the *INF*β promoter in that the constitutive nucleosome-free region at *GRP78* encompasses the TATA box and TIS (Figures 2 and 3). It is similar to what has been shown for the *GCY1* gene in yeast [10], making it one of the first examples of such a promoter in mammals. Since *GRP78* is a single copy gene in the human genome and is essential for cell survival [30 and unpublished data], nucleosome depletion at the core promoter region may have evolved to allow a swift response to ER stress as a protective measure.

The underlying mechanism of nucleosome depletion from promoters is not completely understood. Proposed mechanisms include recruitment of remodeling complexes by activator proteins to cause histone dissociation and nucleosome sliding [32–35] and binding by TBP or other transcription factors, which generates a bent structure in DNA [36–38]. Additionally, the existence of poly(dA:dT) tracts in the promoter region or other properties of the DNA sequence have been proposed to cause a rigid structure of the DNA which disfavors nucleosome positioning [39]. At the *GRP78* core promoter, nucleosome depletion was found in all molecules analyzed and was not correlated with or dependent on TATA box, TIS, or ERSE binding. Additionally, it was not dependent on the remodeling complex BRG/BRM, as no nucleosomes were detected on the *GRP78* core promoter in BRG/BRM-deficient cell lines (A427, SW13) (unpublished data). Also, no poly(dA:dT)-rich flanks of DNA were found within the nucleosome-depleted region. Analysis of the genomic region surrounding the promoter using an algorithm that calculates the probability of nucleosome occupancy at the studied region revealed nearly zero probability for nucleosome positioning at the *GRP78* core promoter (E. Segal, J. Widom, personal communication). Thus, while experimental proof is still wanted, nucleosome depletion might result from a general property of the DNA sequence of the *GRP78* promoter similar to what has recently been suggested for nucleosome-free promoters in yeast [40].

The uniqueness of the M-SPA method lies in its single molecule resolution and the maintenance of promoter integrity, combined with computational analysis. These features enabled us to define functional modules on single molecules in an unsupervised manner and to study their combinatorial organization during the transcription process. We find that at the population level, stress induction results in an overall decrease of nucleosomal patches downstream of the TIS. This may reflect remodeling of nucleosomes in this region consistent with previous studies showing H4 acetylation and arginine methylation at the promoter region after stress induction [23]. However, at the single molecule level, our current data do not show specific correlation between binding of factors at the core promoter region and nucleosome protection downstream of the TIS (Figure 4B). Studying the nucleosome-free region itself using unsupervised clustering analysis reveals the existence of six major modes of the promoter implying a high level of constraint and regulation imposed on it during the activation process. While all clusters are found at all time points analyzed, temporal enrichment analysis reveals that specific clusters are overrepresented or underrepresented at specific phases of induction, allowing us to determine the chronological order of the suggested modes and reveal the nature of these constraints (Figure 6). First, even prior to activation the TATA box is protected to a significant extent, probably by TBP and other factors (Figure 6A). This is true, regardless of activator binding at the ERSEs or TIS occupation and thus does not support a dominant model in which TBP recruitment is dependent on activator binding. As TBP recruitment to the promoter is considered a rate-limiting step for transcription activation, its binding to a large fraction of the promoters might support basal levels of expression and enable a prompt response to an activator signal at the induced state.

After stress induction, our data suggest that protection at the ERSEs increases in a sequential fashion from ERSE1 (closest to the TATA box) to ESRE3 (Figure 6B−6D). While, the three ERSE elements are very similar in sequence and are all needed for optimal stress induction [26,41], slight differences exist between them, which confer different binding affinities for the specific transcription factor complexes. For instance, ERSE1 and ERSE2 have a high affinity binding site for NF-Y [26], while ERSE3 has the best consensus site for YY1 and was shown to be important for YY1-mediated induciblity [42]. YY1 itself acts in concert with stress-induced ATF6 for optimal induction of *GRP78* [23] and ATF6-mediated stimulation is dependent on high affinity NF-Y binding [26]. Thus, it may be speculated that complex binding at ERSE1 may stabilize binding at ERSE2 and ERSE3, facilitating the full induction potential of the promoter.

The binding of factors to the ERSEs facilitates recruitment of the general transcription machinery to the TIS (Figure 6E) which in turn is followed by release of ERSEs' bound factors from the promoter (Figure 6F). This might be a consequence of an active mechanism by which a signal is transduced from the recruited or modified RNA pol II causing the disassembly of the transcription factors from the promoter. Such a hypothesis would be in agreement with the findings that inhibiting phosphorylation of a promoter-bound RNA pol II inhibited the eviction of the activator from the promoter [43].

To conclude, understanding the delicate interplay resulting in transcriptional activation remains a major challenge. The results presented contribute to the emerging view of heterogeneity among activation processes in different types of promoters. *GRP78,* representing a class of genes, which show basal activity together with sharp induction upon stress, may have evolved to allow a specialized regulatory regimen, allowing both a rapid response and a sustained high level of expression during a long period of stress. As shown here, extending the analysis of promoter dynamics from experiments focusing on the average activity of a single factor to experiments studying the entire promoter footprints of single molecules can provide novel insights into nucleosomal organization and transcription factor binding to form a coherent model of gene regulation.

## Materials and Methods

### Cell culture, TG treatment.

The normal human fibroblast LD419 cell line was cultured and maintained as previously described [44]. Stress induction was achieved by TG (Sigma, St. Louis, Missouri, United States) treatment at a final concentration of 300 nM for 0.5 to 16 h as indicated in the text.

### RT-PCR.

Total RNA was extracted from cultured LD419 cells using TRIzol reagent (Invitrogen, Carlsbad, California, United States), according to the manufacturer's instructions. Total RNA (2 to 5 μg) was used for RT. Following incubation with DNase I (Invitrogen), to eliminate possible DNA contamination, Superscript III (Invitrogen) and random hexamers (Promega, Madison, Wisconsin, United States) were used for first-strand cDNA synthesis. Quantitative RT-PCR was performed using an Opticon light cycler with SYBR green I (Sigma), using gene specific primers for *GRP78* [45]. All values were normalized to GAPDH expression ratios.

### ChIP.

ChIP analyses using anti histones/modified histones antibodies were performed as previously described [46]**.** Briefly, uninduced or TG-induced LD419 cells were crosslinked with 1% formaldehyde for 10 min at 37 °C, washed twice with ice-cold PBS, lysed, and sonicated. For transcription factor ChIP, cells were crosslinked with 1% formaldehyde for 15 min at 37 °C, washed twice with ice-cold PBS, once with buffer A (20 mM HEPES [pH 7.6], 0.25% Triton X-100, 10 mM EDTA, 0.5 mM EGTA), and once with buffer B (50 mM HEPES [pH 7.6], 150 mM NaCl, 1 mM EDTA, 0.5 mM EGTA). Washings with buffers A and B consisted of a 10-min rotation and a 7-min spin at 500 × *g* (1,200 rpm) at 4 °C. The resulting nuclei were then lysed using ChIP lysis buffer and sonicated. For both histone and transcription factor, ChIP sonication was performed, taking care that the bulk of DNA fragments was on the order of 200 bp. Antibodies used were 5 μg of anti total histone H3 (Abcam, Cambridge, Massachusetts, United States), 5 μg of anti–H3Ac-K9/K14 (Upstate Biotechnologies, Charlottesville, Virginia, United States), 8 μg of anti–RNA-pol II CTD (UPSTATE BIOTECHNOLOGIES), 15 μg of anti-TBP, 6 μg of anti–NF-Y, and 10 μg of anti-ATF6 (Santa Cruz Biotechnology, Santa Cruz, California, United States). Quantitative analyses were done by real-time PCR (Opticon, Orangeburg, New York, United States) using SYBR green I (Sigma). Primer sequences are listed in Table 1 (R1–R4).

**Table 1:**
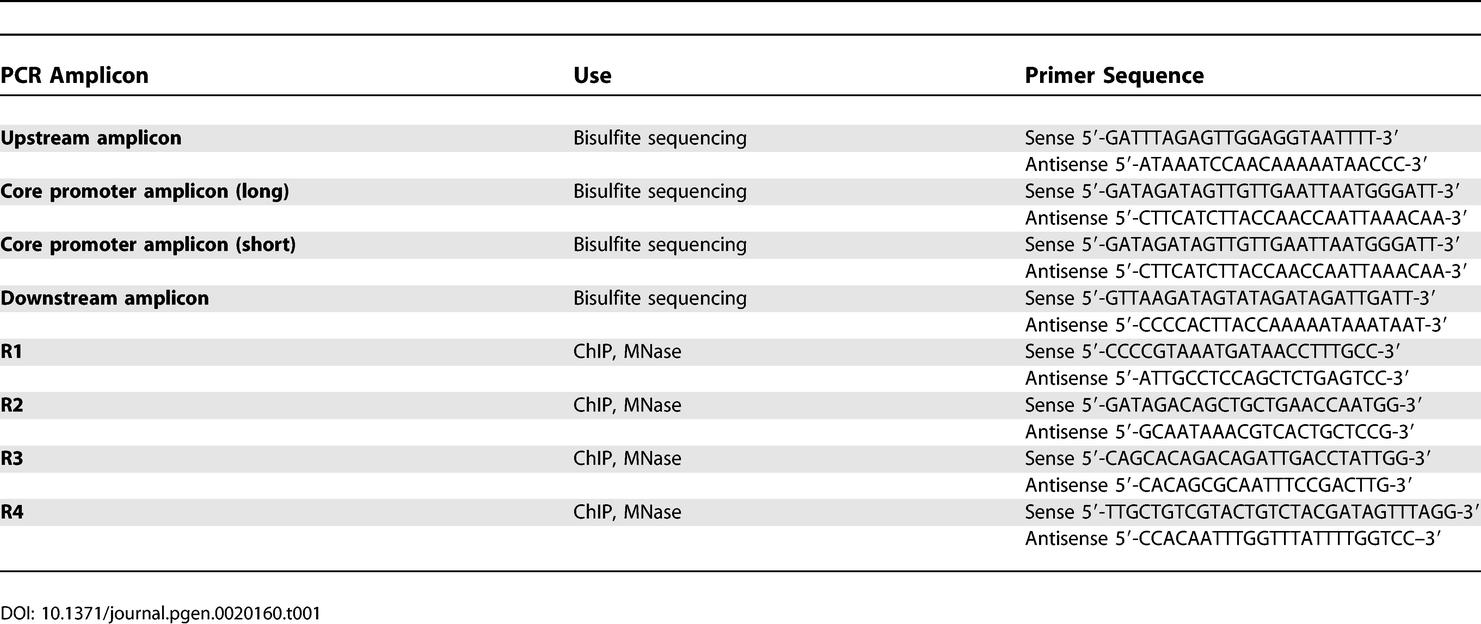
Primer Sequences Used in This Study

### Nuclei extraction.

All procedures for nuclei isolation were performed at 4 °C. Actively growing cells (10^8^ for MNase and DNase I treatments and 10^7^ for M.SssI treatments) were trypsinized and washed twice with ice-cold PBS. Cells were resuspended in 1 ml of RSB buffer (10 mM Tris-HCl [pH 7.4], 10 mM NaCl, 3 mM MgCl_2_) and kept on ice for 10 min. Following this incubation, 0.1 ml of 10% Nonidet P-40 (NP-40) detergent was added and cells homogenized with 10 to 15 strokes of the tight pestle of a Dounce homogenizer. This was followed by two washes with RSB buffer (for MNase and DNase I treatments) or one wash with RSB buffer followed by one wash with M.SssI buffer (for M.SssI treatment, 10 mM Tris-HCl [pH 7.9], 50 mM NaCl, 10 mM MgCl_2_, 1 mM dithiothreitol, and 0.3 M sucrose).

### Nucleosomal DNA preparation and analysis.

Extracted nuclei (1.5 × 10^7^) were resuspended in 200 μl of 1× MNase reaction buffer (10 mM Tris-HCl [pH 7.4], 10 mM NaCl, 3 mM MgCl_2_ 0.25 M sucrose, 100 μM PMSF, 3 mM CaCl_2_) and incubated with MNase for 15 min at 37 °C. The reactions were stopped by the addition of EDTA/EGTA (up to 10 mM each). After centrifugation the nuclear pellet was resuspended in RSB buffer containing 5 mM EDTA/EGTA to obtain soluble chromatin which was fractionated through sucrose gradient centrifugation (in 5% to 25% sucrose, 10 mM Tris-HCl [pH 7.4], 0.25 mM EDTA, 200 mM PMSF, 100 mM NaCl) at 30,000 rpm for 16 h at 4 °C. As a control, we used naked DNA partially digested with MNase. Naked and nucleosomal DNA samples were treated with RNase and proteinase K followed by phenol/chloroform extraction and ethanol precipitation and subjected to quantitative analysis by real-time PCR (Opticon) using SYBR green I and the primers listed in Table 1 (R1–R4).

### M.SssI treatments.

Nuclei extracted as described above were resuspended in 1× M.SssI buffer to a concentration of 10^7^ nuclei/0.1 ml and incubated with M.SssI immediately after preparation for 15 min at 37 °C. The methylation reactions were carried out in 1× M.SssI buffer with 160 μM SAM (supplied with M.SssI by New England Biolabs, Beverly, Massachusetts, United States). Nuclei from 10^6^ cells (approximately 6 μg of DNA) or 6 μg of naked DNA in a total reaction volume of 150 μl were treated with 60 U of M.SssI. Reactions were stopped by the addition of an equal volume of stop solution (20 mM Tris-HCl [pH 7.9], 600 mM NaCl, 1% SDS, 10 mM EDTA, 600 μg/ml proteinase K). Samples were incubated at 55 °C for 3 h followed by 37 °C overnight and the DNA purified by phenol/chloroform extraction and ethanol precipitation.

### DNase I footprinting.

Nuclei extracted as described above, or naked DNA, were resuspended in RSB buffer (10 mM Tris-HCl [pH 7.4], 10 mM NaCl, 3 mM MgCl_2_) plus 0.25 M sucrose. These were then digested at 37 °C for 15 min using various concentrations of DNase I (Worthington, San Francisco, California, United States) to obtain a suitable range of digestion of genomic DNA as revealed by EtBr staining. Digested genomic DNA was purified, redigested by RsaI, resolved on a 1.5% agarose gel, and Southern blotted. The blot was hybridized with a 220-bp PCR-amplified RsaI probe located at −955 to −735 bp relative to the *GRP78* transcription start site. Primer sequences used for probe amplification were forward, 5′-ATC AGA CCT TTT CCT GGA ATG-3′, and reverse, 5′-CAA AGG AAT TGC CTC CAG C-3′.

### Methylation analysis using bisulfite genomic sequencing.

Bisulfite genomic sequencing was used to analyze the methylation patterns of individual DNA molecules. The *GRP78* promoter was PCR amplified using DNA that had undergone M.SssI treatment followed by sodium bisulfite conversion according to standard protocols [47,48]. PCRs on the bisulfite converted DNA were performed using primers complementary to the deaminated DNA (listed in Table 1, Upstream amplicon, Core promoter amplicon long, Core promoter amplicon short, Downstream amplicon). For each amplicon, at least two PCR products were cloned into the pCR2.1 vector provided by TOPO-TA Cloning Kit (Invitrogen) and transformed into Escherichia coli following the manufacturer's instructions. The positive screened colonies contained the unique sequence of one individual DNA molecule. Plasmid DNA from the selected positive colonies was purified using the Qiagen plasmid Mini Kit and sequenced at the region of interest. Sequencing was performed at the USC/Norris Microchemical Sequencing Core Facilities. With the exception of fully unmethylated or fully methylated molecules, only molecules with unique modification patterns are shown and were included in the analysis to avoid biases introduced by multiple cloning of the progeny of individual DNA molecules. Of the 372 technically successful sequences that were performed (including the upstream, downstream, and core amplicons), 12 were removed due to nonsufficient conversion (less than 97% conversion efficiency) and 23 sequences were removed from the various reactions due to 100% identity to other sequences derived from the same cloning reaction (see Table S1 for a summary of the sequencing data for each amplicon).

### Footprint modules identification.

To identify footprint modules, we compiled a set of CpGs' footprint binary vectors by pooling data from all time points. We analyzed the similarity among footprints by computing Spearman correlation coefficients and defined a sequence module as a connected component in the graph formed by associating all pairs of CpGs with footprint correlation larger than 0.5 (in other words, we performed single linkage clustering with Spearman correlation cutoff of 0.5). Importantly, under this definition, all modules represent maximal contiguous promoter subsequences, allowing us to use this simple approach and still obtain spatially organized modules. To define the module's activity profile, we averaged the protection status of all modules' CpGs over all sequences taken from a particular time point. To assess the significance of increased/decreased protection levels in different time points, we performed a χ^2^ test comparing the inferred modules' binary states in the samples from one time point to those of the other time points.

### Combinatorial analysis.

To identify combinatorial patterns of protection at the core promoter, we performed standard k-mean clustering on the set of available protection footprints (this time clustering sequences rather than CpGs). We obtained similar cluster structure using several alternative definitions of similarity; results reported here were generated using Pearson correlation. To test for enrichment of specific clusters in samples from specific time points, we performed a standard hypergeometric (Fisher exact) test, assessing the probability of observing a large intersection between the set of sequences in a given cluster and the set of sequences from a given experimental time point.

## Supporting Information

Figure S1 Methylation Status of Naked DNA at the *GRP78* Promoter Region The diagram on top, drawn to scale, represents the region analyzed and indicates the distribution density of the 73 CpG sites (tick marks) included in this region. The positions of the ERSEs (E1, E2, E3), TATA box (T), and transcription initiation site (bent arrow) are indicated. Bisulfite genomic sequencing was performed on the three regions of the promoter covering 1,100 bp, before (A) and after (B) 15 min of M.SssI treatment of naked genomic DNA. Each horizontal line with a string of circles represents the methylation profile for one DNA molecule. White circles indicate unmethylated, and black circles, methylated, CpG sites.
(180 KB PDF)

Figure S2 Partial Methylation of Naked DNA at the Core Promoter Region Does Not Reveal Defined Footprints The diagram on top, drawn to scale, represents the region analyzed and indicates the distribution density of the 37 CpG sites included in this region. The TIS (bent arrow), TATA box (T), and ERSE elements (E1–E3) are marked. Depicted are 40 sequences (top) derived from naked DNA which was treated with M.SssI to achieve average partial methylation (60%) similar to that of the pool of 294 sequenced molecules derived from M.SssI-treated nuclei. Each horizontal line with a string of circles represents the methylation profile for one DNA molecule. White circles indicate unmethylated, and black circles, methylated, CpG sites. The bottom graph shows the summed methylation at the distinct CpG sites.
(80 KB PDF)

Table S1 Summary of Bisulfite Sequencing Data for the Various Amplicons (26 KB DOC)

## Abbreviations

bp: base pair(s)
CHiP: chromatin immunoprecipitation
ER: endoplasmic reticulum
ERSE: endoplasmic reticulum stress response element
M-SPA: methylase-based single promoter analysis
TBP: TATA-binding protein
TG: thapsigargin
TIS: transcription initiation site
