# Supplementary material for: Constitutive Nucleosome Depletion and Ordered Factor Assembly at the GRP78 Promoter Revealed by Single Molecule Footprinting
Source: PLoS Genet. 2006 Sep 22;2(9):e160. doi: 10.1371/journal.pgen.0020160 (PMC1574359; doi:10.1371/journal.pgen.0020160)

Figure S1

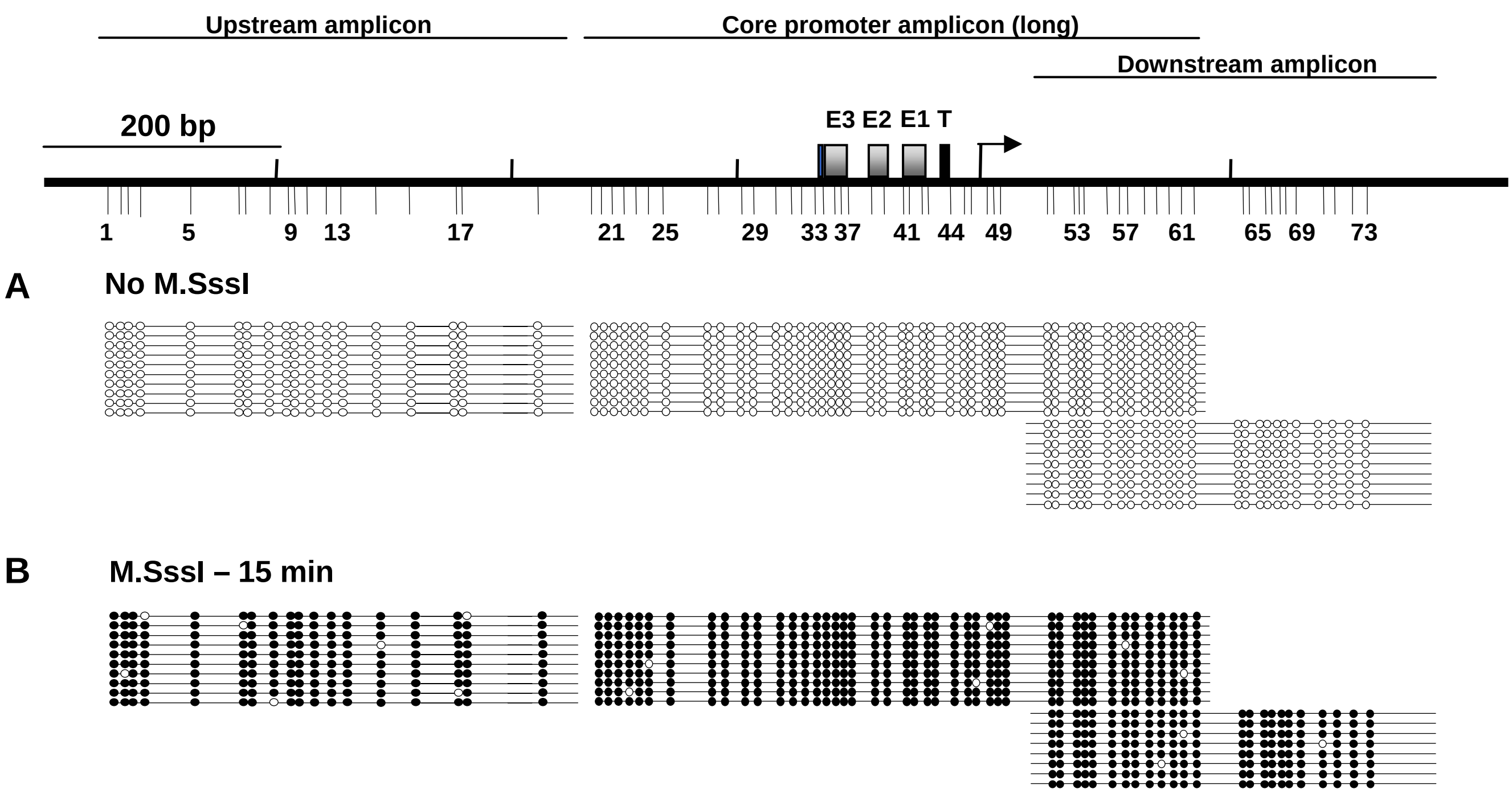

Upstream amplicon
Core promoter amplicon (long)
Downstream amplicon
200 bp
E3 E2 E1 T
1 5 9 13 17 21 25 29 33 37 41 44 49 53 57 61 65 69 73
A
No M.SssI
B
M.SssI – 15 min

Supplementary Figure 2

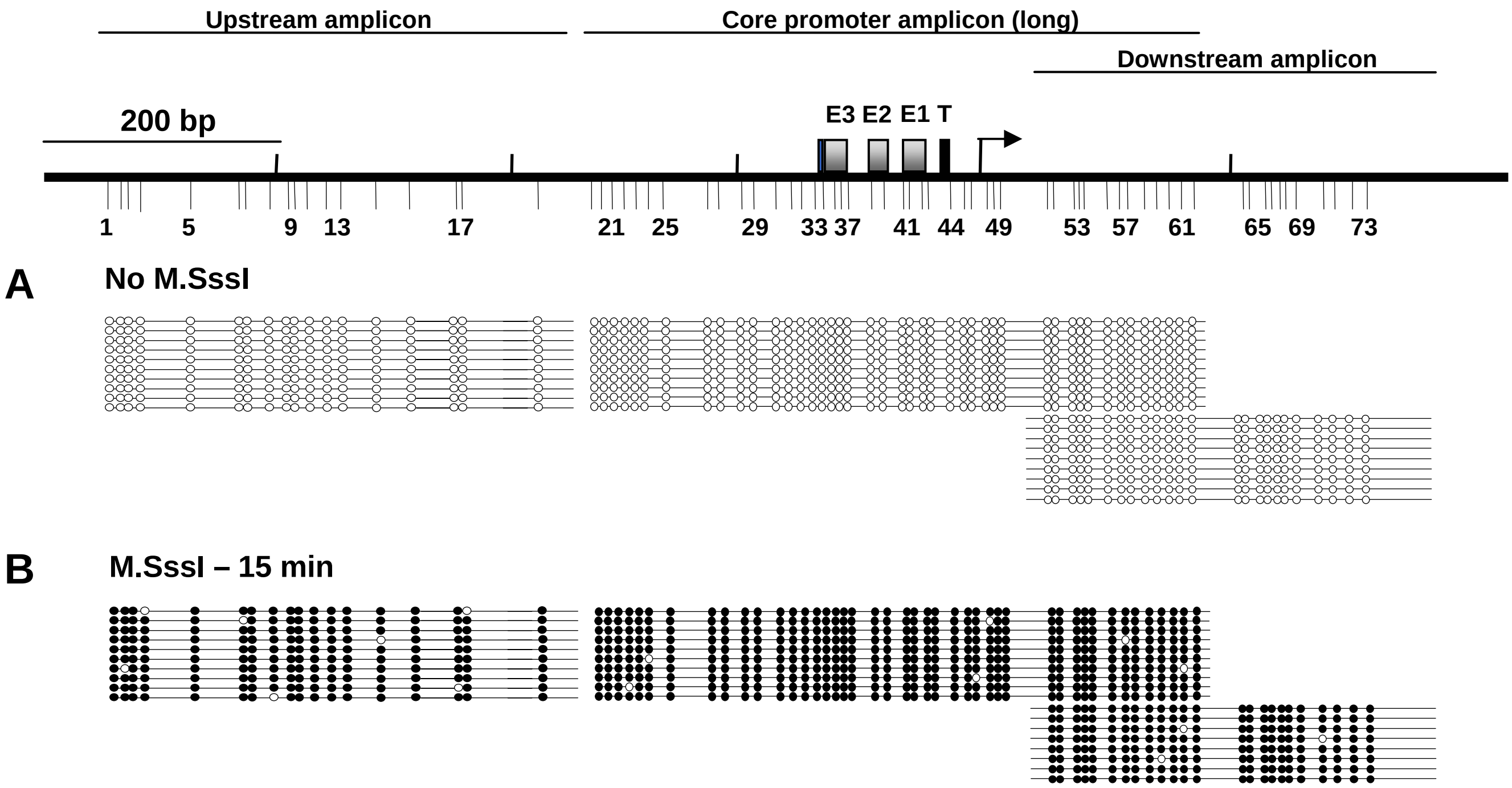

Supplement: Supplementary file 1 [file pgen.0020160.sg001.pdf]
