# Supplementary material for: Constitutive Nucleosome Depletion and Ordered Factor Assembly at the GRP78 Promoter Revealed by Single Molecule Footprinting
Source: PLoS Genet. 2006 Sep 22;2(9):e160. doi: 10.1371/journal.pgen.0020160 (PMC1574359; doi:10.1371/journal.pgen.0020160)
Supplement: Supplementary file 2 [file pgen.0020160.sg002.pdf]

Figure S2

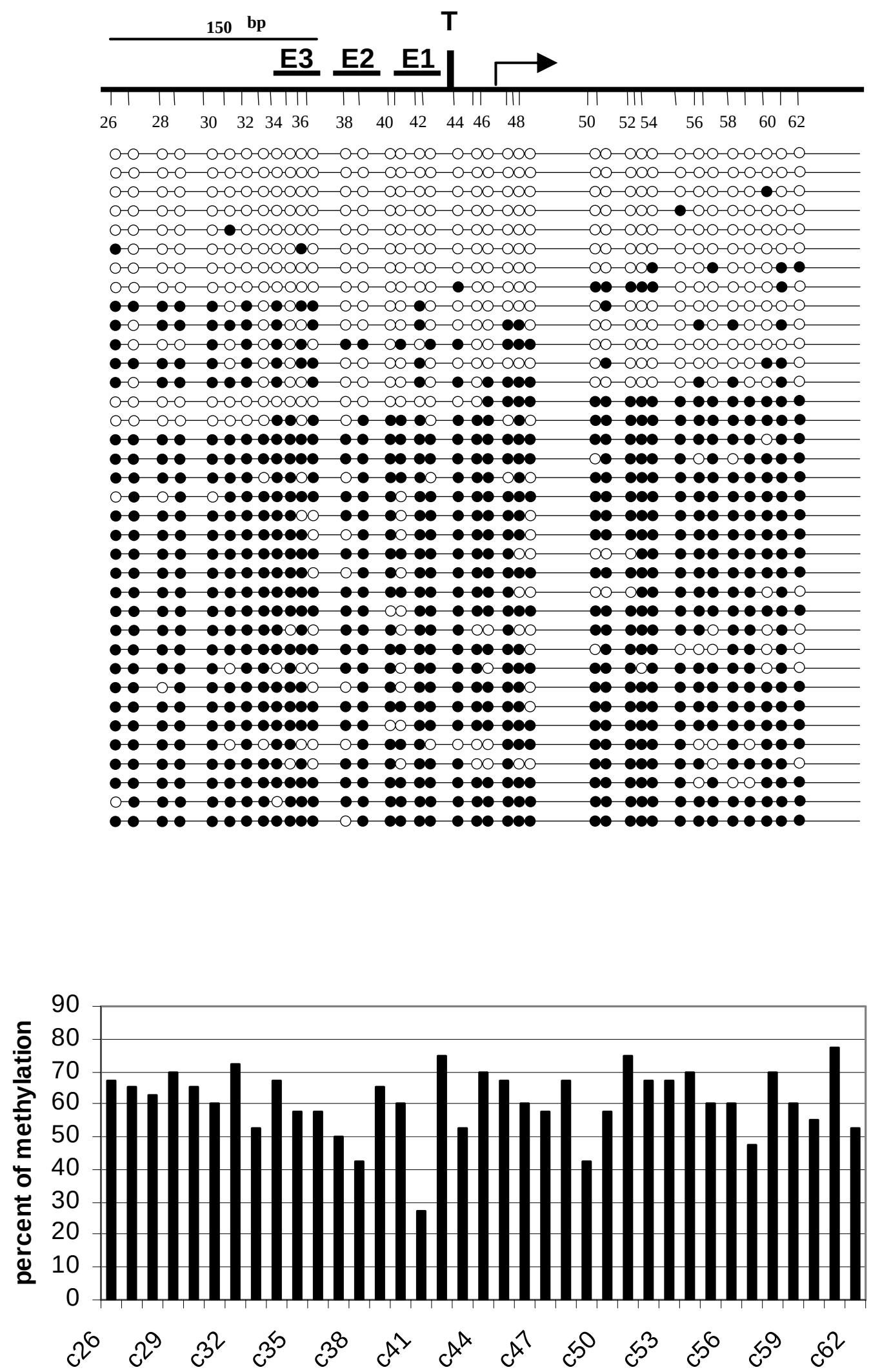

150 bp
T
E3
E2
E1
26
28
30
32
34
36
38
40
42
44
46
48
50
52
54
56
58
60
62
90
80
70
60
50
40
30
20
10
0
percent of methylation
c26
c29
c32
c35
c38
c41
c44
c47
c50
c53
c56
c59
c62
