# Supplementary material for: Constitutive Nucleosome Depletion and Ordered Factor Assembly at the GRP78 Promoter Revealed by Single Molecule Footprinting
Source: PLoS Genet. 2006 Sep 22;2(9):e160. doi: 10.1371/journal.pgen.0020160 (PMC1574359; doi:10.1371/journal.pgen.0020160)
Supplement: Supplementary file 3 [file pgen.0020160.st001.doc]

**Table S1. Summary of bisulfite sequencing data for the various amplicons**

| **Amplicon** | **Total number of sequences** | **Sequences removed due to non sufficient conversion (< 97%)** | **Sequences removed due to identical cloning** | **Sequences analyzed** |
| --- | --- | --- | --- | --- |
| **Upstream amplicon** | 25 | 1 | 1 | 23 |
| **Core promoter amplicon (short and long combined)** | 327 | 11 | 22 | 294 |
| **Downstream amplicon** | 20 | 0 | 0 | 20 |
| **Total** | 372 | 12 | 23 | 337 |
